# Plasma fibrinolysis is related to the degree of organ dysfunction but not to the concentration of von Willebrand Factor in critically ill patients

**DOI:** 10.1186/1477-9560-7-10

**Published:** 2009-06-19

**Authors:** Karim Zouaoui Boudjeltia, Sandra Ollieuz, Michael Piagnerelli, Patrick Biston, Philippe Cauchie, Jean-Louis Vincent, Dany Brohee, Michel Vanhaeverbeek

**Affiliations:** 1 Experimental Medicine Laboratory (ULB 222 Unit), CHU-Charleroi, Vesale Hospital, 6110-Montigny-le-Tilleul, Belgium; 2 Department of Intensive Care, CHU-Charleroi, 6000-Charleroi. Belgium; 3 Department of Intensive Care, Erasme University Hospital, Université Libre de Bruxelles, 1070-Brussels. Belgium

## Abstract

**Background:**

Endothelial cell dysfunction, by promoting fibrin deposition, has been implicated in the development of multiple organ failure. Altered fibrinolysis during inflammation may participate in microvascular alterations. We sought to determine whether plasma fibrinolysis was related to the severity of organ dysfunction and/or to the levels of von Willebrand factor (vWF antigen), as a marker of endothelium dysfunction, in critically ill patients.

**Methods:**

Forty-nine consecutive patients admitted to an adult medico-surgical intensive care unit (ICU) with (18) or without sepsis (31) were included. C-reactive protein and vWF levels were measured on ICU admission and plasma fibrinolysis was assessed by the Euglobulin Clot Lysis Time (ECLT). The sequential organ failure assessment (SOFA) score and the simplified acute physiology score (SAPS) II were calculated on admission.

**Results:**

ECLT was significantly longer in septic than in non-septic patients [1033 min (871–1372) versus 665 min (551–862), p = 0.001]. There were significant correlations between ECLT and C-reactive protein (CRP) concentrations (r = 0.78, p < 0.001) and the Sequential Organ Failure Assessment (SOFA) score (r = 0.39, p = 0.006). The level of vWF was not correlated with the ECLT (r = -0.06, p = 0.65) or the SOFA score (r = -0.02, p = 0.88).

**Conclusion:**

ECLT measurement at admission could be a marker of organ dysfunction and a prognostic indicator in critically ill patients.

## Introduction

Endothelial cells (ECs) are in tight contact with all organs, so that EC activation and damage has been implicated in the development of multiple organ failure (MOF) [1]. Among the proposed mechanisms, altered fibrinolysis may promote fibrin deposition and thereby contribute to microvascular alterations [2].

Fibrinolysis and inflammation may be intertwined. Elevated concentrations of C-reactive protein (CRP), especially when they persist over time, are correlated with the risk of MOF and death [3]. CRP may inhibit fibrinolysis by inducing release of plasminogen activator inhibitor-1 (PAI-1) from human aortic ECs [4]. In addition, the administration of recombinant CRP to volunteers increases circulating PAI-1 levels [5]. We previously showed that hypofibrinolysis assessed by the Euglobulin Clot Lysis Time (ECLT) was strongly correlated with CRP concentrations in critically ill patients [6].

ECLT is the test most commonly used to estimate plasma fibrinolytic capacity. The ECLT result represents the balance between tissue plasminogen activator (t-PA) and PAI-1 activities. We sought to define the relationship between ECLT, as a marker of fibrinolysis, von Willebrand factor (vWF), a marker of endothelial dysfunction [7], and the severity of the clinical syndrome in critically ill patients. Organ dysfunction was estimated by the Sequential Organ Failure Assessment (SOFA) score [8] and the risk of hospital mortality by the simplified acute physiologic score (SAPS II) [9].

## Materials and methods

### Subjects

The study protocol was approved by the ethics committee of A. Vésale hospital and informed consent was obtained from each patient or their closest relative. Over a 5 month period, we enrolled all consecutive critically ill patients at ICU admission. Sepsis was defined by usual criteria [10]. Exclusion criteria were: age < 18 years, transfusion of red blood cells or other blood components in the 72 h prior to study entry, active bleeding, haematological disorders, cytotoxic chemotherapy in the 6 months prior to study entry, burns, cardiogenic shock, cirrhosis, or pregnancy. The SAPS II score and SOFA scores were determined on admission.

### Blood samples

In each patient, blood samples were drawn once during the 24 hours following ICU admission; serum and plasma samples were obtained from the same venipuncture. Serum samples were collected in vacuum tubes without anticoagulant. Plasma samples were harvested in citrated vacuum tubes and put in melting ice. Whole blood was collected in EDTA-treated tubes. CRP was measured by antibody-binding and turbidity measurement on SYNCHRON LX^® ^(Analys, Belgium). Fibrinogen was determined by the Clauss method on a STA^® ^automate (STAGO, Paris, France). An immuno-turbidimetric assay for vWF determination was used (Liatest^® ^vWF:Ag, STAGO, Paris France). Leucocytes and platelet counts were determined using a haemocytometer (CELL-DYN4000^®^, Abbott, Belgium).

### Plasma fibrinolytic capacity

ECLT was measured on fresh plasma using a method described elsewhere [11]. Briefly, we designed a completely computerized, semi-automatic, 8-channel device for measurement and determination of fibrin clot lysis (Lysis Timer, EREM, Belgium). The lysis time is evaluated by mathematical analysis of the lysis curve and the results are expressed in minutes (range: 5 to 9999). The efficiency scores of the method are < 4% for intra-assay and < 7% for inter-assay. Three hundred microlitres of acetic acid (0.25%) and 3.6 ml of desionized water are added to 400 μl plasma (final pH≈5.9). The sample is then put into melting ice for 20 min and centrifuged at 4000 g for 10 min at 4°C. The supernatant is discarded and the pellet is resuspended in 400 μl of Owren-Koller buffer (DIAGNOSTICA STAGO^®^, France). Clot formation starts when 100 μl of thrombin (1.75 U/ml, DIAGNOSTICA STAGO^®^) are added. Normal values are median 208 min (range, 118–303; n = 25) for men and median 117 min (range, 100–174; n = 21) for premenopausal women [11].

### Statistics

We used SigmaStat^® ^3.5 (SPSS). The data are presented as median value and range [25%–75%]. Correlations between variables were analyzed using a Spearman correlation test. A multiple logistic regression model was used for survival analysis. A probability level of p < 0.05 was considered as statistically significant.

## Results

Of the 49 patients, 18 had sepsis (11 pneumonia, 2 peritonitis, 2 skin infection, 1 urinary tract infection, 1 endocarditis and 1 mediastinitis); the 31 other patients had exacerbated chronic obstructive pulmonary disease (COPD) without evidence of sepsis (n = 21), postanoxic coma or stroke (n = 6), or heart failure (n = 4). Mortality was higher in septic than in non-septic patients (55% vs. 35.4%).

The biological characteristics of the patients are shown in Table 1. Inflammatory variables, such as fibrinogen and CRP levels, were higher in the septic than in the non-septic patients. ECLT was also higher in the septic than in the non-septic patients. There was no significant difference in vWF between septic and non-septic patients.

**Table 1:** Population characteristics.

Parameters	Values (all patients, n = 49)	Non sepsis n = 31	Sepsis n = 18	p
SAPS II	52.5 (45–63)	48 (38.5–64)	54 (49.2–62)	0.21
SOFA	7 (5.7–10)	6.5 (5–9)	8 (6–10.2)	0.41
ECLT (min)	791 (609–1023)	665 (551–862)	1033 (871–1372)	**< 0.001**
vWF (%)	368 (293–490)	370 (289–450)	367 (294–513)	0.53
WBC (10^3^/μl)	11 (8–14)	12.1 (8.3–14.3)	9.43 (7.7–16.3)	0.9
Haemoglobin (g/dl)	10.3 (8.7–12.2)	10.7 (9.4–12.7)	9.7 (8–11.2)	**0.03**
Haematocrit (%)	32.5 (27.2–37.1)	32.7 (29.3–38.5)	31.7 (25.2–33)	**0.06**
Activated clotting time (sec)	42 (35–48)	37.5 (32.5–45.5)	48 (45–51)	**< 0.001**
Fibrinogen (g/l)	4.8 (3.73–6)	4.2 (3.5–5.08)	6.1 (4.6–8.1)	**0.002**
Prothrombin time (%)	70 (57–77)	73 (65–82)	61 (48.5–71.2)	**0.006**
Platelet count (10^3^/μl)	70.5 (57.5–77)	250 (141–290)	267 (194–322)	0.27
Total bilirubin (mg/dl)	0.62 (0.41–0.91)	0.7 (0.42–0.96)	0.58 (0.39–0.85)	0.52
C-reactive protein (mg/dl	9.37 (4.4–23.4)	6.44 (2.8–11.8)	24.5 (15.5–34.6)	**< 0.001**
Urea (mg/dl)	62.3 (40.05–78.15)	59 (39.6–75.1)	74 (44–78)	0.27
Creatinine (mg/dl)	1.02 (0.65–1.57)	1.01 (0.6–1.5)	1.3 (0.65–1.83)	0.59
ICU mortality	22 (42.3%)	11 (35.4%)	11 (55.5%)	**0.05**

Data shown as median (25%–75%), except for ICU mortality which is shown as absolute n (%). ECLT: Euglobulin Clot Lysis Time; WBC: White blood cells; SOFA: sequential organ failure assessment; SAPS: simplified acute physiology score; vWF: von Willebrand factor

**Figure 1:**
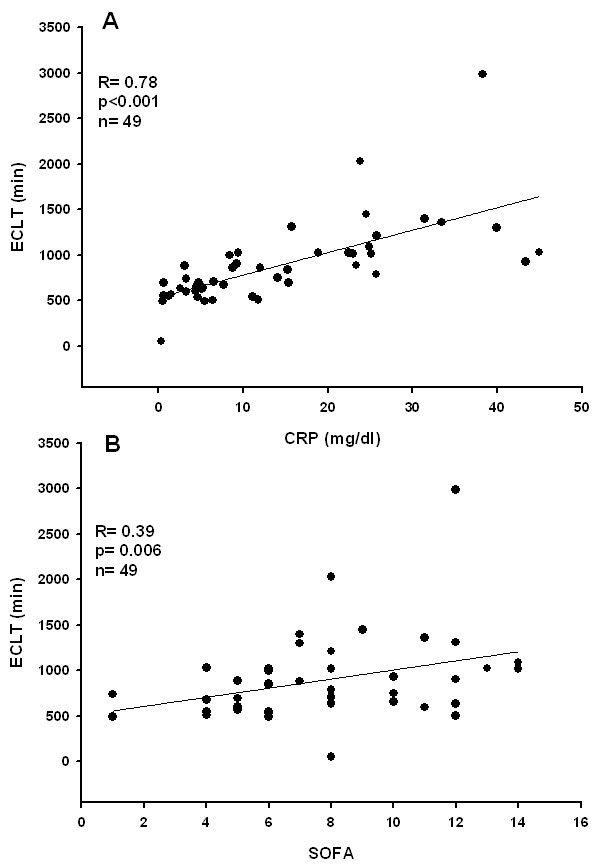
**Relationships between CRP, SOFA and ECLT (A and B respectively)**.

In the whole population, there was a significant correlation between ECLT and CRP levels (n = 49, r = 0.678, p < 0.001, fig 1A) and between ECLT and SOFA score (r = 0.39, p = 0.009, fig 1B). There was a weak correlation between vWF and CRP levels (r = 0.29; p = 0.04), but not between vWF and either ECLT (r = -0.06, p = 0.65) or the SOFA score (r = -0.02, p = 0.88).

Table 2 shows the correlations between SOFA score, SAPS II score, vWF levels and ECLT in non-septic and septic patients. The relationship between SOFA score and ECLT remained significant in the non-septic patients (r = 0.45, p = 0.01) and was almost significant in the septic patients (r = 0.39, p = 0.11). Other correlations were not significant.

**Table 2:** correlations between SOFA, SAPS and ECLT.

Correlations	Non-sepsis n = 31	Sepsis n = 18
SOFA/ECLT	0.45 (0.01)	0.39 (0.11)
SAPS/ECLT	0.12 (0.54)	-0.13 (0.61)
SOFA/vWF	0.1 (0.58)	-0.009 (0.96)
SAPS/vWF	0.06 (0.76)	-0.13 (0.62)
ECLT/vWF	-0.18 (0.32)	0.007 (0.97)

Spearman Rank correlations: R (p)

A multiple logistic regression model was used to evaluate the ICU death event (Table 3). The SAPS II (p = 0.048) and ECLT (p = 0.049) were retained in the multiple logistic regression as explanatory variables for ICU mortality.

**Table 3:** Multiple logistic regression evaluating ICU mortality.

Variables	Coef (B)	Std error	Wald X^2 ^(p)	OR	95%CI
Intercept	-6.6	2.98			
Sepsis	-1.19	1.09	1.19 (0.27)	0.3	0.03–2.58
SOFA	-0.16	0.20	0.62 (0.43)	0.85	0.57–1.27
SAPS II	0.09	0.05	3.89 (**0.048**)	1.105	1.001–1.21
ECLT	0.04	0.002	3.87 (**0.049**)	1	1–1.009
vWF	-0.0002	0.002	0.004 (0.94)	1	0.99–1.005
CRP	-0.03	0.04	0.4 (0.52)	0.96	0.88–1.068

## Discussion

Several studies have reported a relationship between coagulation abnormalities, organ dysfunction and mortality in critically ill patients [12-16], but the role of fibrinolysis has not been well studied. Fibrinolytic activity is primarily determined by the balance between t-PA and PAI-1 levels. ECs are responsible for the production and release of t-PA and contribute to the release of PAI-1. Multiple factors, including lipoproteins, cytokines and inflammatory markers, modulate EC production of t-PA and PAI-1 [17]. There are several reasons why decreased fibrinolysis could be considered as a surrogate marker of EC dysfunction, organ failure and mortality [18-20]. Indeed, the deposition of fibrin on confluent ECs causes EC aggregation and disorganization of the monolayer, thus increasing permeability [21,22]. In addition, fibrin is also a potent stimulus for EC production and release of IL-8, a leucocyte chemotactic factor [23].

In a large retrospective analysis of 1789 ICU patients, Okabayashi et al [12] reported that the SOFA score was correlated with antithrombin concentrations (r = 0.32, p < 0.05), prothrombin fragment concentration (r = 0.4, p < 0.001) and fibrinolysis as assessed by t-PA-PAI-1 complexes (r = 0.52, p < 0.001). Mavrommatis et al [24] studied the activation of fibrinolysis in septic patients during the first day in the ICU, and observed that t-PA/PAI-1 complexes (4.7 ± 0.6 vs 2.1 ± 0.2 ng/mL) and the percentage of fibrinogen/fibrin degradation products (100 vs 57%) were higher in patients with septic shock than in those without (all p < 0.001). These authors also observed a greater activation of fibrinolysis, as reflected by decreased plasminogen activity (41.6 ± 3.3 vs 87.4 ± 3.2%) and increased t-PA and PAI-1 concentrations [24].

ECLT is a global test for fibrinolysis, representing the balance between t-PA and PAI-1 activities. Previously considered as an imprecise method, we have been able to improve the precision and reproducibility of the test with a new semi-automatic device [11]. Moreover, in contrast to that of vWF (12 h) [25], the half-lives of t-PA (3–4 min) and PAI-1 (10 min) are very short, suggesting that ECLT could be an instantaneous marker of clinical status. We previously showed that ECLT was related to the number of cardiovascular risk factors (hypertension, smoking habit, diabetes, history of coronary event or stroke, menopausal status) [26] and suggested that ECLT could be a surrogate marker of endothelial dysfunction.

The present study confirms the previously described [6] relationship between ECLT and CRP concentrations. This relationship could be explained by the simple fact that CRP reflects the severity of sepsis and the risk of organ failure. Alternatively, there may be a more pathophysiologic explanation, as CRP can act directly on the endothelium and modulates fibrinolysis [4,5]. Another mechanism for the prolonged lysis time in septic patients could be related to the effects of systemic inflammation on the liver, causing activation of various cells and release of cytokines influencing the production of fibrinogen and PAI-1 [27,28].

We did not observe, in our population, a significant correlation between ECLT and SAPS II score, as a severity score, but the number of patients was limited and the mortality was quite high in the non-septic group who had significant co-morbidities.

Circulating plasma vWF is derived exclusively from the endothelium (with some minor expression by megakaryocytes). vWF can be produced by ECs when they are stimulated or damaged by various stimuli, including inflammatory cytokines (e.g., interleukin-1 or tumour necrosis factor), hypoxia, thrombin, histamine and leucocyte elastase [29]. Increased levels of vWF are also influenced by substances like adrenaline (epinephrine), vasopressin, and even cyclosporin [29]. Plasma levels of vWF have been considered as a marker of endothelial dysfunction and damage, although this is controversial [30] and results from studies that have correlated plasma levels of vWF, severity of inflammation and patient outcome are inconsistent [30]. Ware et al [31] reported in 559 patients with acute lung injury and/or with acute respiratory distress syndrome that vWF levels were not different in patients with or without sepsis (p = 0.82). In contrast, Kayal et al [32] observed that vWF plasma levels were significantly higher in patients with severe infection (n = 25) than in non-infected patients (n = 7) (p < 0.001). In the present study, there was no significant difference in vWf antigen between septic and non-septic patients. These data confirm that vWF is a somewhat controversial marker of sepsis.

Although, SAPS II and ECLT were retained as explanatory variables of ICU mortality in the multiple logistic regression model, these results must be interpreted carefully in light of the small sample size.

This study has some limitations, including the small sample size and the single-centre nature. Nevertheless, these are interesting data and should be validated in larger heterogeneous populations.

In conclusion, ECLT measurements could represent a marker of alterations in EC function and of organ dysfunction and may be a useful prognostic factor in critically ill patients.

## Abbreviations

COPD: Chronic Obstructive Pulmonary Disease; CRP: C-reactive Protein; ECLT: Euglobulin Clot Lysis Time; ECs: Endothelial Cells; ICU: Intensive Care Unit; PAI-1: Plasminogen Activator Inhibitor-1; SAPS II: Simplified Acute Physiology Score II; SOFA: Sequential Organ Failure Assessment; t-PA: tissue Plasminogen Activator; vWF: von Willebrand Factor

## Competing interests

The authors declare that they have no competing interests.

## Authors' contributions

KZB: Laboratory analysis, writing of the manuscript and design of the study. SO: patient recruitment. MP: writing and analysis of results. PB: recruitment of patients. PC: laboratory analysis. JLV: analysis of the study. DB: coordination and design. MV: statistical analysis and coordination.

All authors read and approved the final manuscript.
